# Oral colonization by *Candida* species in HIV-positive patients: association and antifungal susceptibility study

**DOI:** 10.1590/S1679-45082018AO4224

**Published:** 2018-08-01

**Authors:** Letícia Silveira Goulart, Werika Weryanne Rosa de Souza, Camila Aoyama Vieira, Janaina Sousa de Lima, Ricardo Alves de Olinda, Claudinéia de Araújo

**Affiliations:** 1 Universidade Federal de Mato Grosso, Rondonópolis, MT, Brazil.; 2 Universidade Estadual da Paraíba, Campina Grande, PB, Brazil.

**Keywords:** Candida, Candidiasis, oral, HIV, Microbial sensitivity tests, Antifungal agents, Candida, Candidíase bucal, HIV, Testes de sensibilidade microbiana, Antifúngicos

## Abstract

**Objective:**

To investigate antifungal susceptibility and factors associated with oral colonization by *Candida* species in HIV-positive patients.

**Methods:**

A prospective study based on convenience sampling of subjects recruited from a pool of confirmed HIV-positive individuals seen at a specialty outpatient service in Rondonópolis, Mato Grosso, Brazil). Oral swabs were collected from 197 patients. *Candida* species were identified by standard microbiological techniques (phenotypic and molecular methods). Antifungal susceptibility was investigated using the broth microdilution method.

**Results:**

A total of 101 (51.3%) patients were *Candida* spp carriers. *Candida albicans* was the most prevalent species (80%). Patients aged 45 to 59 years (Prevalence ratios: 1.90; 95%CI: 1.57-6.31) and 60 years or older (Prevalence ratios: 4.43; 95%CI: 1.57-34.18) were at higher risk of oral colonization by *Candida* species. Resistance to fluconazole and ketoconazole, or to itraconazole, corresponded to 1% and 4%, respectively.

**Conclusion:**

Age (45 years or older) was the only factor associated with oral colonization by *Candida* . Low rates of antifungal resistance to azoles were detected in yeast isolates obtained from HIV-positive patients. Findings of this study may contribute to proper therapeutic selection for oral candidiasis in HIV-positive patients.

## RESUMO

**Objetivo:**

Investigar a suscetibilidade a antifúngicos e os fatores associados à colonização oral por espécies de *Candida* isoladas de pacientes HIV positivo.

**Métodos:**

Estudo prospectivo realizado com amostragem por conveniência de indivíduos HIV positivo, acompanhados por um serviço de atendimento especializado da cidade de Rondonópolis, Mato Grosso, Brasil. Foram coletados *swabs* orais de 197 pacientes. As espécies de *Candida* foram identificadas por técnicas microbiológicas fenotípicas padrão e por método molecular. A suscetibilidade antifúngica foi determinada pelo método de microdiluição em caldo.

**Resultados:**

Cento e um (51,3%) pacientes foram colonizados por *Candida* spp. *Candida albicans* foi a espécie mais prevalente (80%). Identificou-se um maior risco de colonização oral por espécies de *Candida* em pacientes com idade entre 45 e 59 anos (razão de prevalência: 1,90; IC95%: 1,57-6,31) e 60 anos ou mais (razão de prevalência: 4,43; IC95%: 1,57-34,18). A resistência ao fluconazol e ao cetoconazol foi de 1% cada e de 4% ao itraconazol.

**Conclusão:**

O único fator associado à colonização oral por espécies de *Candida* foi ter 45 anos ou mais. Identificamos baixa taxa de resistência antifúngica aos azóis entre as leveduras isoladas de pacientes HIV positivo. Estes achados podem contribuir para selecionar o tratamento da candidíase oral em pacientes HIV positivos.

## INTRODUCTION

Roughly 40.6 thousand cases of acquired immunodeficiency syndrome (AIDS) have been reported annually in Brazil over the last five years.^(^ ^1^ ^)^ AIDS is caused by the human immunodeficiency virus (HIV) and is characterized by reduced CD4 T-cell counts and increased patient susceptibility to opportunistic infections due to impaired immune response.^(^ ^2^ ^)^ Specific oral manifestations play a significant role in diagnosis and monitoring of disease progression.^(^ ^3^ ^)^

Oropharyngeal candidiasis is one of the first clinical signs of AIDS, affecting 50 to 95% of HIV-positive individuals.^(^ ^4^ ^)^ *Candida* species are commensal microorganisms of the oral mucosa; however, in the presence of predisposing factors, these may become pathogenic and cause infection.^(^ ^5^ ^)^ Several factors are thought to predispose to oral candidiasis, such as extremes of age, dental prosthesis, smoking and salivary, hormone, nutritional or immunological changes.^(^ ^6^ ^)^ Oral candidiasis may be pseudomembranous, erythematous, hyperplastic or mucocutaneous, or manifest as angular cheilitis^.(^ ^7^ ^)^ *Candida albicans* is responsible for most episodes of oral candidiasis, but other species, such as *Candida glabrata* , *Candida krusei* , *Candida tropicalis* , *Candida parapsilosis* and *Candida dubliniensis* , are often implicated.^(^ ^8^ ^)^

Intrinsic and acquired ( *i.e* ., treatment-induced) antifungal resistance in *Candida species* have a negative impact on disease management.^(^ ^9^ ^)^ For this reason, standardized antifungal susceptibility testing methods were developed by the European Committee on Antimicrobial Susceptibility Testing (EUCAST) and the Clinical and Laboratory Standards Institute (CLSI).^(^ ^10^ ^)^ These tests play an increasingly significant role in therapeutic decision making and drug development studies, and can be used to monitor antifungal resistance development in epidemiological investigations.^(^ ^9^ ^,^ ^11^ ^)^

## OBJECTIVE

To investigate antifungal susceptibility and factors associated with oral colonization by *Candida* species isolated from HIV-positive patients.

## METHODS

A prospective study with HIV-positive individuals seen at the specialty service of the *Secretaria Municipal de Saúde* of Rondonópolis, Mato Grosso, Brazil. Participants were recruited via convenience sampling during routine medical appointments, between January and May 2015. All participants were informed about the study objectives, risks and benefits and signed an Informed Consent Form. This study was approved by the Research Ethics Committee of *Hospital Universitário Júlio Muller* , *Universidade Federal de Mato Grosso* (UFMT), committee opinion no. 749,382, CAAE: 31905114.6.0000.5541. Patients aged under 18 years were excluded. Data on age, sex, antiretroviral medication, previous opportunistic infections, concurrent sexually transmitted diseases, intravenous drug use and CD4 T-cell counts were extracted from medical records.

### Yeast isolation and identification

Oral swabs collected from HIV-positive patients were seeded onto Sabouraud Dextrose agar (Difco, Detroit, USA) supplemented with chloramphenicol (100μg/mL) and chromogenic medium CHROMagar *Candida* (PROBAC, São Paulo, Brazil). Plates were incubated at 37°C for 48 to 72 hours and yeast species confirmed via species-specific polymerase chain reaction (PCR), as described by Liguori et al.^(^ ^12^ ^)^ DNA extraction was achieved using a DNA extraction kit (Mobio, Carlsbad, CA, USA), according to manufacturer’s instructions. Polymerase chain reaction was carried out in a total volume of 25μL; reactions contained 10mM Tris-HCl (pH 8.3), 50mM KCl, 1.5mM MgCl2, 0.38mM of deoxyribonucleotide triphosphate (0.2mM each), 3.2mM primers and 1.25U TaqDNA polymerase. Oligonucleotides CA ( *C. albicans* , 5’- TCA ACT TGTCAC AGA TTA TT-3 ‘), CGLA ( *C. glabrata* , 5’- CAC GAC TCGACA CTT TCT AAT T-3’), CT ( *C. tropicalis* , 5’-AAG AAT TTAACG TGG AAA CTT A-3 ‘), CK ( *C. krusei* , 5’- GAT TTA GTA CTACAC TGC GTC A-3’) and ITS4 (5’- TCC TCCGCT TAT TGA TAT GC-3’) were used. Amplification reactions were carried out using the following parameters: initial denaturation (92°C for 2 minutes), 35 denaturation cycles (95°C for 1 minute), annealing (50°C for 1 minute), extension (72°C for 1 minute) and final extension (72°C for 10 minutes).

### Antifungal susceptibility testing

Antifungal susceptibility of isolates was determined using broth microdilution, according to CLSI M27-A3 standards.^(^ ^13^ ^)^ Antifungal agents were diluted in RPMI-1640 medium with MOPS buffer (Sigma ChemicalCo., USA) at pH 7.0. Drugs were distributed into 96-well microplates at final 0.03 to 16μg/mL (itraconazole and ketoconazole) or 0.125 to 64μg/mL (fluconazole) concentrations. Microdilution plates were incubated at 35°C and inspected within 24 to 48 hours to determine the minimum inhibitory concentrations (MIC), or the lowest concentration required to inhibit fungal growth by ≥50% compared to positive controls. Findings were expressed in terms of MIC variation (MIC50 or MIC90, growth inhibition in 50% and 90% of isolates, respectively).

Epidemiological cutoff values for antifungal susceptibility testing (CLSI M27-S4 guidelines)^(^ ^14^ ^)^ are as follows: fluconazole susceptibility of *C. albicans* and *C. tropicalis -* MIC ≤2μg/mL sensitive, ≥8μg/mL resistant, 4μg/mL dose-dependent susceptibility (DDS); fluconazole susceptibility of *C. glabrata –* MIC ≤32μg/mL DDS, MIC ≥64μg/mL resistant. *C. krusei* isolates and thought to be intrinsically resistant to fluconazole, therefore respective MICs should not be interpreted using this scale. Reference values of *Candida* species susceptibility to itraconazole correspond to MIC ≤0.125μg/mL (sensitive), ≥1μg/mL (resistant) and 0.25 to 0.5μg/mL DDS.^(^ ^15^ ^)^ Reference values for ketoconazole were not included in CLSI guidelines; therefore, parameters given by Mulu et al.,^(^ ^15^ ^)^ were adopted (MIC ≥4μg/mL equals resistance).

### Data analysis

Multivariate analysis was performed using a logistic regression model to investigate factors associated with oral colonization by *Candida* species. Prevalence ratios (PR), 95% confidence intervals (95%CI) and p values were calculated for different factors. The level of significance was set at 5%. Statistical analyses were performed using R software.

## RESULTS

This study included 197 HIV-positive patients (99 men) aged between 19 and 78 years (mean age of 42.1 years). Sociodemographic and clinical features of participants are presented in table 1 . Most (n=193, 98%) patients had received highly active antiretroviral therapy (HAART) for five years on average; the most common (52.8%) therapeutic regimen consisted of a combination of nucleoside and non-nucleoside reverse transcriptase inhibitors. Patient history analysis revealed 78 (39.6%) cases of opportunistic infections, with candidiasis accounting for most episodes, followed by herpes-zoster (11.2% and 10.6%, respectively). Concurrent sexually transmitted infections were diagnosed in 17.8% of patients. Intravenous drug use was reported by 14% of participants. CD4 T-cell counts ranged from 16 to 2,299 cells/mm^3^ (mean count, 663 cells/mm^3^).

**Table 1:** Demographic and clinical characteristics of HIV-positive patients

Characteristics	Colonization	

	Positive (n= 101) n (%)	Negative (n = 96) n (%)
Sex		
Male	42 (41.6)	57 (59.3)
Female	59 (58.4)	39 (40.7)
Age, years		
19-29	17 (16.8)	15 (15.6)
30-44	43 (42.6)	46 (46.9)
45-59	30 (29.7)	32 (33.4)
≥60	11 (10.9)	3 (3.1)
Antiretroviral regimen		
PI+NRTI	47 (46.5)	41 (42.7)
NRTI+NNRTI	49 (48.5)	55 (57.3)
Duration of antiretroviral therapy, years		
0-5	57 (56.4)	47 (49)
6-11	30 (29.7)	29 (30.2)
≥12	14 (13.9)	20 (20.8)
History of opportunistic infection	40 (39.6)	38 (39.6)
Other sexually transmitted infection	17 (16.8)	18 (18.7)
Use of intravenous drugs	16 (10.9)	11 (11.5)
CD4 T-lymphocytes (cells/mm^3^)		
<200	14 (13.9)	8 (8.3)
200-700	50 (49.5)	49 (51)
>700	37 (36.6)	39 (40.7)

PI: protease inhibitor; NRTI: nucleoside reverse transcriptase inhibitors; NNRTI: non-nucleoside reverse transcriptase inhibitors.

Oral colonization by *Candida* species was detected in 51.3% of patients, *C. albicans* being the most common species (80%), followed by *C. glabrata* (14%), *C. tropicalis* (4%) and *C. krusei* (2%).

Logistic regression results are given in table 2 . Colonized and non-colonized patients did not differ significantly with regard to sex (p=0.3760), duration of antiviral therapy (p=0.6820), antiviral regimen (p=0.405), history of opportunistic infections (p=0.392), concurrent sexually transmitted infections (p=0.718) or intravenous drug use (p=0.413). CD4 T-cell counts were not correlated with the presence of *Candida* species in the oral cavity of HIV-positive patients. Age was the only risk factor for oral colonization by *Candida* spp.; colonization was associated to patients aged 45 to 59 years (PR: 1.90; 95%CI: 1.57-6.31) and 60 years or more (PR: 4.43; 95%CI: 1.57-34.18), and colonization risks increased with age.

**Table 2:** Prevalence ratios of oral colonization by *Candida* species

Factors	PR	95%CI	p value*
Sex			
Male		1 (reference)	0.3760
Female	1.43	0.65-3.13	
Age, years			
19-29		1 (reference)	0.0273
30-44	1.04	0.36-2.97	
45-59	1.90	1.57-6.31	
≥60	4.43	1.57-34.18	
Antiretroviral regimen			
PI+NRTI		1 (reference)	0.405
NRTI+NNRTI	0.73	0.35-1.53	
Duration of antiretroviral therapy, years			
0-5		1 (reference)	0.6820
6-11	0.89	0.37-2.12	
≥12	0.61	0.2-1.84	
History of opportunistic infection	0.72	0.34-1.53	0.392
Other sexually transmitted infection	1.19	0.47-2.98	0.718
Use of intravenous drugs	0.60	0.18-2.05	0.413
CD4 T-lymphocytes (cells/mm^3^)			
<200		1 (reference)	0.718
200-700	0.6	0.17-2.09	
>700	0.68	0.18-2.48	

* Logistic regression. 95%CI: 95% confidence interval; PR: prevalence ratios; PI: protease inhibitor; NRTI: nucleoside reverse transcriptase inhibitors; NNRTI: non-nucleoside reverse transcriptase inhibitors.

Testing of *Candida* spp. isolates revealed 84% sensitivity, 15% DDS and 1% resistance to fluconazole; 99% sensitivity and 1% resistance to ketoconazole; and 73% sensitivity, 23% DDS and 4% resistance to itraconazole. One isolate ( *C. albicans* ) was resistant to fluconazole, one ( *C. tropicalis* ) to ketoconazole and four (two *C. glabrata* , one *C. albicans* and one *C. tropicalis* ) to itraconazole *.* MIC50 and MIC90 values for fluconazole, ketoconazole and itraconazole corresponded to 0.5, 0.03 and 0.125 *μ* g/mL, and 0.5, 0.03 and 0.5 *μ* g/mL, respectively ( Table 3 ).

**Table 3:** Antifungal susceptibility to *Candida* species in HIV-positive patients

Species (n)	Antifungal	MIC range	MIC50	MIC90	Sensitive	DDS	Resistant
		(µg/mL)	(µg/mL)	(µg/mL)	n (%)	n (%)	n (%)
*Candida albicans* (n=81)	Fluconazole	0.125-8	0.125	0.125	79 (98)	1 (1)	1 (1)
	Cetoconazole	0.03-2	0.03	0.03	81 (100)	0 (0)	0 (0)
	Itraconazole	0.03-4	0.125	0.5	65 (81)	15 (18)	1 (1)
*Candida glabrata* (n=14)	Fluconazole	0.25-4	0.5	2	0 (0)	14 (100)	0 (0)
	Cetoconazole	0.03-0.5	0.03	0.5	14 (100)	0 (0)	0 (0)
	Itraconazole	0.06-2	0.125	2	7 (50)	5 (36)	2 (14)
*Candida tropicalis* (n=4)	Fluconazole	0.125-0.5	0.125	0.125	4 (100)	0 (0)	0 (0)
	Cetoconazole	0.03-16	0.06	0.125	3 (75)	0 (0)	1 (25)
	Itraconazole	0.03-16	0.25	0.5	1 (25)	2 (50)	1 (25)
*Candida krusei* (n=2)	Fluconazole	0.25	0.25	0.25	-	-	-
	Cetoconazole	0.03	0.03	0.03	2 (100)	0 (0)	0 (0)
	Itraconazole	0.125	0.125	0.125	1 (100)	1 (100)	0 (0)
Total (n=101)	Fluconazole	0.125-8	0.125	0.5	83 (84)	15 (15)	1 (1)
	Cetoconazole	0.03-16	0.03	0.03	100 (99)	0 (0)	1 (1)
	Itraconazole	0.03-16	0.125	0.5	74 (73)	23 (23)	4 (4)

MIC: minimum inhibitory concentration; DDS: dose-dependent sensibility.

## DISCUSSION

Oral colonization by *Candida* species is common in HIV-positive individuals^(^ ^16^ ^)^ and affected 51.3% of patients in this sample. Similar findings have been reported in studies carried out in China (49.5%),^(^ ^17^ ^)^ Brazil (50.4%),^(^ ^18^ ^)^ Taiwan (51.4%)^(^ ^19^ ^)^ and Nigeria (52.5%).^(^ ^20^ ^)^ Identification of asymptomatic carriers of *Candida* spp. is important for identification of prevalent species in epidemiological studies, and may assist therapeutic decision making. However, oral colonization should not be investigated in routine medical practice, given the lack of clinical significance and potential generation of unnecessary costs.

*C. albicans* was the prevailing species (80%) in this group of patients, while *C. glabrata* was the most common non- *albicans* species. Prevalence of these microorganisms in the oral mucosa of patients with HIV/AIDS has been reported elsewhere.^(^ ^18^ ^,^ ^21^ ^,^ ^22^ ^)^ *C. albicans* is the most common species isolated from the oral mucosa of HIV-positive individuals, with prevalence ranging from 70 to 82.1%.^(^ ^23^ ^-^ ^26^ ^)^ *C. glabrata* has emerged as a significant pathogen, particularly in the oral mucosa, either as a co-infecting agent associated with *C. albicans* or a sole species isolated from oral lesions. Oropharyngeal infections associated with *C. glabrata* tend to be more severe and refractory to treatment compared to candidiasis caused by *C. albicans* alone.^(^ ^17^ ^,^ ^21^ ^,^ ^22^ ^,^ ^27^ ^)^

Factors potentially associated with oral colonization by *Candida* spp *.* in HIV-positive patients were analyzed in this study. Patients aged 45 years or over were at increased risk of yeast colonization, and risks increased with age. Positive associations between increased risks of oral colonization by *Candida* spp. and age in HIV-positive patients undergoing HAART were described by Esebelahie et al.,^(^ ^20^ ^)^ with higher prevalence rates between 61 and 70 years. Correlations between the presence of *Candida* in the oral cavity of HIV-positive individuals and age were also demonstrated by Kantheti et al.^(^ ^28^ ^)^ In that study,^(^ ^28^ ^)^ risks were identified in non-HAART treated patients aged 41 to 50 years and HAART treated patients aged 51 to 60 years. More frequent use of dental prosthesis in middle-aged and elderly patients may explain the increased risk of *Candida* spp. colonization and infection in these age groups.^(^ ^17^ ^)^

Protease inhibiting antivirals revolutionized AIDS treatment, with significant reduction in opportunistic infection rates, particularly candidiasis.^(^ ^29^ ^)^ Infection attenuation may reflect not only improved immunological status but also direct inhibition of aspartic proteases in *Candida* spp.^(^ ^30^ ^)^ Protease inhibitors block aspartic protease expression *in vivo* and promote fungal biotype selection, affecting *Candida* spp. prevalence and susceptibility to antifungal agents.^(^ ^31^ ^)^ Similar to other trials,^(^ ^17^ ^,^ ^26^ ^,^ ^32^ ^,^ ^33^ ^)^ *Candida* ssp. carrier state was not significantly associated with protease inhibitor-based antiretroviral therapy in this study.

CD4 T-cell counts were not associated with the presence of yeasts in the oral mucosa of patients. Similar findings have been described in previous studies reporting equivalent CD4 T-cell counts in HIV-positive patients with and without oral colonization by yeasts.^(^ ^16^ ^,^ ^20^ ^,^ ^32^ ^,^ ^34^ ^)^ However, CD4 T-cell counts below 200 cells/mL are thought to be a risk factor for *Candida* spp. colonization.^(^ ^19^ ^,^ ^26^ ^,^ ^27^ ^)^

Antifungal susceptibility testing permits accurate treatment selection and provides significant contributions to the understanding of local and global fungal resistance epidemiology.^(^ ^12^ ^)^ In this trial, testings performed using the broth microdilution method revealed low prevalence of oral *Candida* spp. resistance to fluconazole, ketoconazole and itraconazole (1%,1% and 4% respectively). Low rates of yeasts resistance to fluconazole (0.7%),^(^ ^32^ ^)^ ketoconazole (1.5%)^(^ ^35^ ^)^ and itraconazole (4.7%)^(^ ^15^ ^)^ have been reported. Higher resistance to itraconazole compared to the other azoles tested in this analysis supports findings of previous studies.^(^ ^17^ ^,^ ^35^ ^,^ ^36^ ^)^ Resistance to azolic compounds in *Candida* is often attributed to selection pressures exerted by antifungal agents in response to exposure of oral candidiasis patients to repeated, short- or long-term suppressive therapy.^(^ ^15^ ^)^ Treatment of candidiasis remains challenging to date. Antifungal susceptibility testing should precede antifungal therapy whenever possible.^(^ ^7^ ^)^

## CONCLUSION

*Candida albicans* was the most prevalent *Candida* species in the oral mucosa of HIV-positive patients in this sample. Individuals aged 45 years or older were at greater risk of oral colonization by *Candida* species. Most isolates were susceptible to azolic antifungal agents. Findings of this study emphasize the relevance of accurate molecular identification of *Candida* species for proper therapeutic agent selection in patients with oral candidiasis.
